# FKBP51 is associated with early postoperative cognitive dysfunction in elderly patients undergoing hip fracture surgery

**DOI:** 10.1097/MD.0000000000014037

**Published:** 2019-02-01

**Authors:** Li-Wei Wang, Mei-Jun Zhu, Yan Li, Sheng-Tao Wang, Mei-Yan Zhou, You-Jia Yu, Zheng-Liang Ma

**Affiliations:** a Department of Anesthesiology, Nanjing Drum Tower Hospital Clinical College of Nanjing Medical University, Nanjing; b Department of Anesthesiology, Xuzhou Central Hospital, Xuzhou; c Department of Anesthesiology, Suzhou Xiangcheng People's Hospital, Suzhou, Jiangsu; d Department of Pain, Shandong Provincial Hospital Affiliated to Shandong University, Jinan, Shandong, China.

**Keywords:** FKBP51, glucocorticoid receptor, inflammation, postoperative cognitive dysfunction

## Abstract

Enhanced inflammation response was increasingly reported in association with postoperative cognitive dysfunction (POCD). Glucocorticoid receptor (GR) signal plays a key role in suppression of inflammation. This prospective cohort study aimed to evaluate GR signaling in elderly patients undergoing selective operation.

One hundred twenty-six elderly patients were scheduled for hip fracture surgery with general anesthesia. Plasma cortisol levels and the expression levels of GR and FK506 binding protein 51 (FKBP51) in leukocytes were determined at 1 day preoperatively and 7 days. Postoperatively postoperative pain was assessed following surgery using visual analog pain scale (VAS). Neuropsychological tests were performed before surgery and 1 week postoperation. A decline of 1 or more standard deviations in 2 or more tests was considered to reflect POCD.

POCD incidence in participants was 28.3% at 1 week after surgery. POCD patients presented significantly higher cortisol and FKBP51 levels compared with non-POCD patients (*P* < .05). Compared with non-POCD patients, VAS scores at 12 hours after surgery were higher in POCD patients (*P* < .05). No significant difference in expression levels of GR was found between groups POCD and non-POCD patients.

High expression of FKBP51 in leukocytes and glucocorticoid resistance were associated with POCD in aged patients following hip fracture surgery.

## 1 Introduction

Postoperative cognitive dysfunction (POCD) is a severe postoperative complication, which involves a wide range of cognitive functions including working memory, long-term memory, information processing, attention, and cognitive flexibility.^[1]^ POCD is associated with prolonged hospitalization, inability to cope independently and premature unemployment.^[2]^ The mechanism to the development of POCD is poorly understood. The increasing researches suggest that neuroinflammation contributes to the development of POCD. Abnormal enhancement of inflammatory response was found in individuals vulnerable to POCD.^[3–5]^ Usually, inflammation response induced by surgery is transient and self-limited, but enhanced inflammation response after surgery may cause neuron damage and cognition impairment. Glucocorticoid receptor (GR) plays a key role in maintaining moderate inflammation levels. Expression variance of GR caused by all kinds of factors, including FK506 binding protein 51 (FKBP51), affect the function of GR signal,^[6,7]^ which maybe is the underlying reason of the susceptibility to POCD.

This research will differentiate between POCD and non-POCD by neuropsychological tests. Using the methods of molecular biology, we will try to elucidate the relationship of POCD and the expression of GR and FKBP51 in leukocytes. The study may preliminarily provide a universal explanation of pathogenic mechanism of POCD and offer new target and orientation for POCD prevention.

## 2 Methods

### 2.1 Patients

This is a prospective cohort study approved by the Ethics Committee of Xuzhou Central Hospital. Written informed consent was obtained from all patients who underwent total hip-replacement surgery. Our previous study found that POCD incidence was 33.9% in elderly patients undergoing total hip-replacement surgery.^[8]^ With significance set at 0.05 and power set at 0.9, calculated sample size should not be <88 by the use of GPower 3.1.9.2 based on a cohort study. Eligible subjects were American Society of Anesthesiologists (ASA) I or II patients between 65 and 80 years of age, scheduled for total hip-replacement surgery. Exclusion criteria were ASA > II; peptic ulcer disease, cardiac-cerebral vascular disease; history of drug and alcohol abuse; extended glucocorticoid therapy; hepatic and/or kidney dysfunction; body mass index > 35; patients on antidepressants; Mini-Mental State Examination (MMSE) score < 23; and inability to comply with the study protocol or procedures. Patients with psychiatric or neurological disorders, such as depression or insomnia, were excluded, to decrease the likelihood that the disease itself and drugs (i.e., benzodiazepine, antidepressants, etc.) would interfere with evaluation of cognitive function.^[9,10]^

### 2.2 Anesthesia and postoperative treatment

All participants received general anesthesia according to a standardized protocol. Anesthesia was induced with 0.1 mg/kg midazolam, 0.2 mg/kg cisatracurium, 2 mg/kg propofol, and 0.6 mg/kg sufentanil, and maintained with remifentanil and propofol. Bispectral Index Score was maintained at 40 to 60 by adjusting the propofol infusion rate. Heart rate, arterial pressure, PETCO_2_, SpO_2_, body temperature, blood gas tests, and hepatic and kidney dysfunction tests were recorded continuously. The datum deviated from its nominal values was included in the statistics. Patients revived spontaneously without administration of any anesthetic antagonists. Dezocine was used for patient-controlled analgesia (PCA) and tropisetron was administered for nausea treatment after surgery. The analgesic consumption was recorded. Postoperative pain was assessed with a 0 to 10 cm linear visual analog scale (VAS) at 1, 3, 6, 12, 24, and 48 hours after surgery.

### 2.3 Neuropsychological tests

The MMSE for screening of cognitive dysfunction was used in this study. Neuropsychological tests were administered before surgery and at 1 week. An experienced neurologist carried out the neuropsychological tests at both times in tranquil surroundings. The test battery, which included 7 tests with 9 subscales, was designed to measure memory, attention and concentration, and psychomotor skills. The tests included: the Mental Control and Digit Span (forward and backward) subtests of the Wechsler Memory Scale, Visual Retention and Paired Associate Verbal Learning subtests of the Wechsler Memory Scale, Digit Symbol subtest of the Wechsler Adult Intelligence Scale-Revised, Halstead-Reitan Trail Making Test (Part A), and Grooved Pegboard Test (favored and unfavored hand).^[8]^ A decline of 1 or more standard deviations (SDs) in 2 or more tests was considered to reflect POCD, which have been described in our previous study.^[6]^ The patients were divided into the POCD group and the non-POCD group according to neuropsychological tests at 7 days after the operation.

### 2.4 Plasma samples collection and detection

Blood samples were collected immediately before surgery and within 1 week after surgery every morning at 8:00 am. After centrifugation at 2500g for 10 minutes, plasma and leukocytes samples were extracted and stored at 80°C until use. Plasma cortisol levels were measured by radioimmunoassay.

### 2.5 Western blot analysis

Western blotting was used to determine the expression of GR and FKBP51 in the total protein extract from leukocytes 1 day before surgery and to up to 7 days after surgery. Equal amounts of protein were loaded and separated by sodium dodecyl sulfate-polyacrylamide gel electrophoresis and transferred to nitrocellulose membranes. The membranes were blocked in 5% nonfat milk for 2 hours at room temperature and then incubated overnight at 4°C with rabbit anti-GR (1:200), rabbit anti-FKBP51 (1:500), and mouse anti-β-actin (1:1000). After the incubation, the membranes were washed 3 times with phosphate-buffered saline (pH 7.4) containing 0.3% Triton X-100 (PBS-T) and incubated with corresponding secondary antibodies conjugated with horseradish peroxidase (1:500) for 2 hours at room temperature. The protein signals were finally visualized using an enhanced BCIP/NBT Alkaline Phosphatase Color Development Kit.

### 2.6 Quantitative reverse transcriptase polymerase chain reaction

Quantitative reverse transcriptase polymerase chain reaction was used to assess the mRNA levels of GR in leukocytes 1 day before surgery and to up to 7 days after surgery. Total RNA was extracted using a TRIzol reagent kit and transcribed into cDNA using a high-capacity cDNA reverse transcription kit. Real-time PCR analysis was performed using the Roche LightCycler 480 detection system using a SYBR Select Master Mix Kit. The relative expression levels of GR were normalized to GAPDH.

### 2.7 Statistical analysis

All of the data are presented as the mean ± SD of independent experiments. Qualitative variables were statistically analyzed with chi-squared or Fisher exact test. The normality test of the synthetical evaluation index is conducted by using the Kurtosis and Skewness coefficients of the evaluating indexes. Independent-samples *t* test or Mann–Whitney *U* test was used for other quantitative variables. Intragroup comparisons were analyzed by paired-samples *t* tests. Multiple logistic regression analysis was performed to find any potential confounders of POCD. *P* < .05 was considered to be statistically significant.

## 3 Results

### 3.1 Patient characteristics

**Figure 1:**
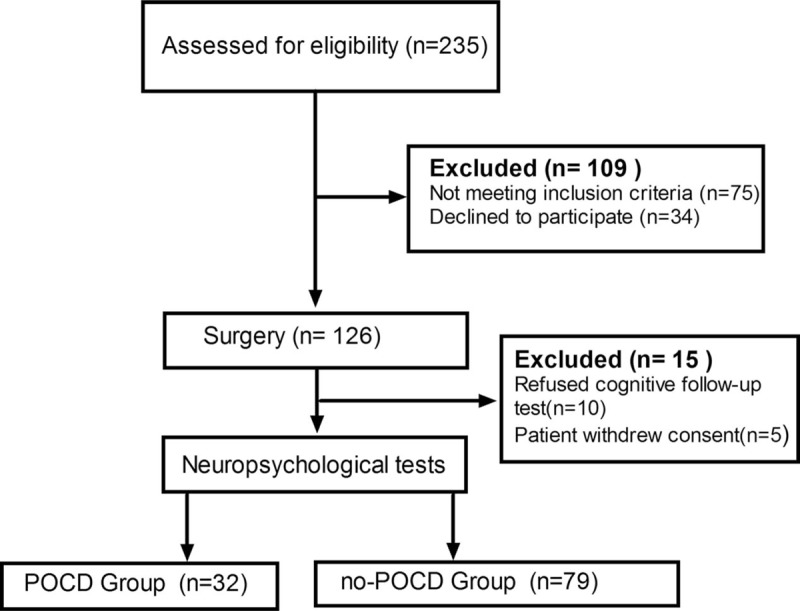
Enrollment flowchart of patients through this study on cognitive dysfunction.

From May 2017 to August 2017, 126 patients were included in the trial. The flow chart of patients through the study and detailed reasons for exclusion are provided in Fig. 1. All the patients underwent the operation successfully. A total of 15 patients were lost to follow-up at 1-week follow-up. The demographic, clinical, and surgical characteristics of patients in POCD and non-POCD groups are presented in Table 1. No significant difference in the demographic and clinical characteristics was observed between the 2 groups. There is no correlation between POCD and any demographic or perioperative factors by multiple logistic regression analysis (Table 2).

**Table 1:**
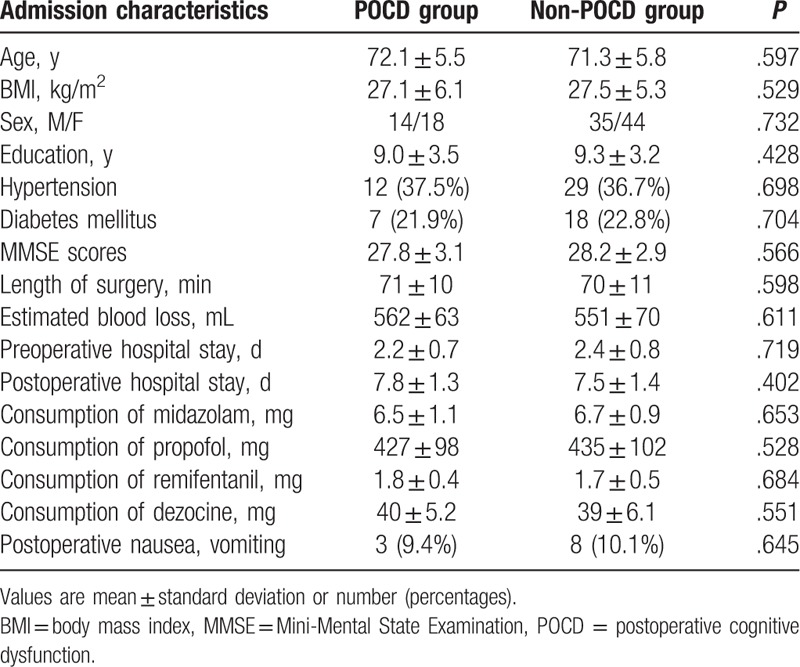
Demographic, clinical, and surgical characteristics.

**Table 2:**
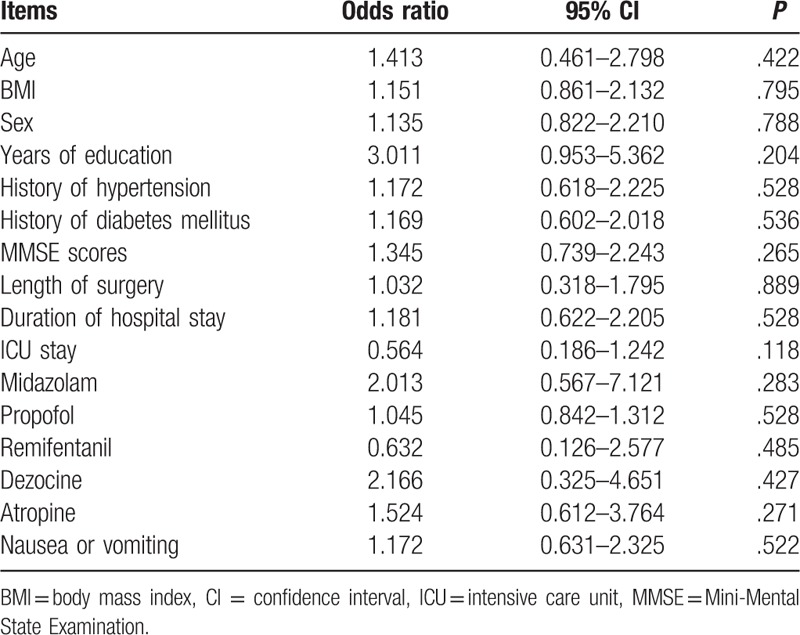
Multiple logistic regression analysis for demographic and perioperative factors.

### 3.2 Neuropsychological test and pain assessment results

At 7 days after surgery, 32 patients fulfilled the diagnostic criteria for POCD. The incidence rate of POCD was 28.8% (32/111) at 7th day postoperatively in this study (Table 3). The mean and SD values of the cognitive parameters in each group are shown in Table 4. There were significant differences in scores of mental control, Digit symbol, and Pegboard favored hand between the 2 groups (*P* < .05). VAS scores in POCD group were higher than that in non-POCD group at 12 hours after surgery (*P* < .05). There was no statistic difference in VAS scores at other times after surgery between the 2 groups (Table 5).

**Table 3:**
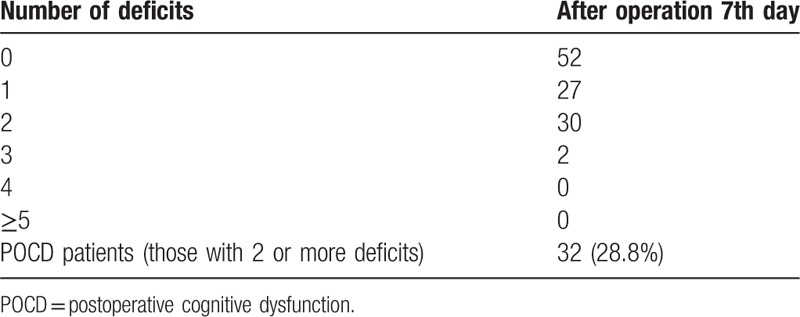
Comparison of occurrence of postoperative neuropsychological deficit.

**Table 4:**
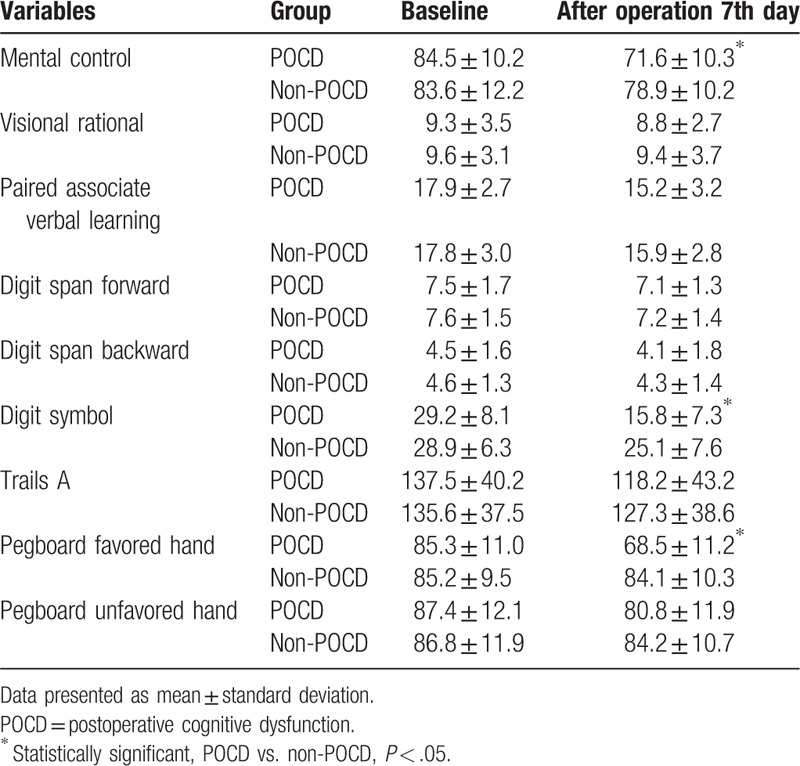
Neuropsychological assessment scores at baseline, 7 days follow-up in patients.

**Table 5:**
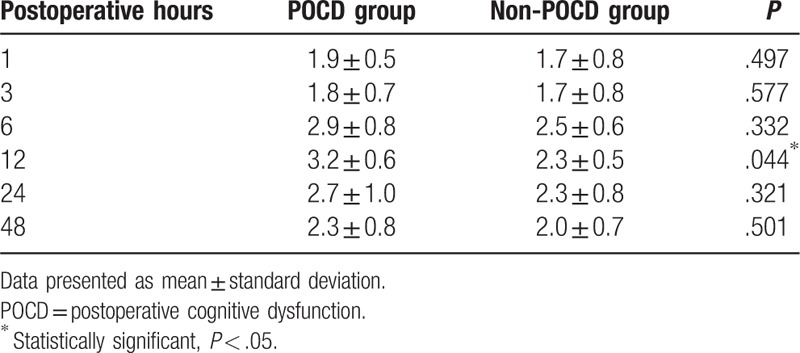
Visual analog scale pain scores.

### 3.3 Plasma cortisol levels and expression of GR and FKBP51

**Figure 2:**
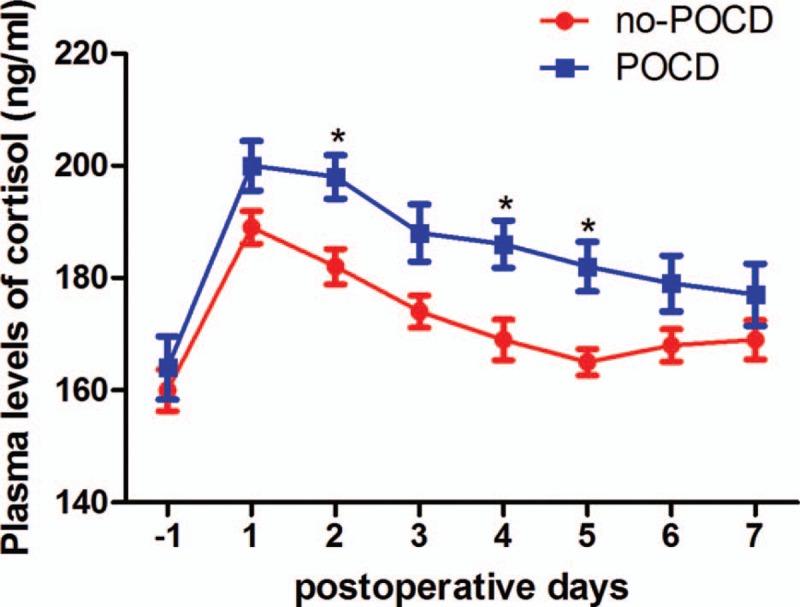
Plasma levels of cortisol before and after surgery in POCD and non-POCD group. POCD = postoperative cognitive dysfunction. ^∗^*P* < .05 vs. non-POCD group.

**Figure 3:**
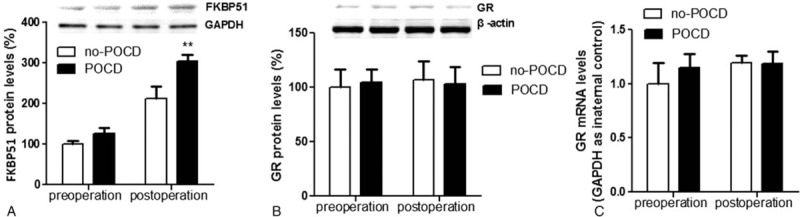
The expression of FKBP51 and GR in leukocytes before and after surgery in POCD and non-POCD group. (A) The levels of FKBP51 in POCD patients were markedly higher than that in non-POCD patients after surgery. No significant difference in expression levels of GR protein (B) and mRNA (C) was found between groups POCD and non-POCD patients (B, C). FKBP51 = FK506 binding protein 51, GR = glucocorticoid receptor, POCD = postoperative cognitive dysfunction. ^∗^*P* < .05 versus non-POCD group.

All patients presented higher cortisol and FKBP51 levels after surgery compared with baseline levels in both groups (*P* < .05). However, the levels of cortisol and FKBP51 in POCD patients were markedly higher than that in non-POCD patients after surgery (Figs. 2 and 3A). No significant difference in expression levels of GR was found between groups POCD and non-POCD patients (Fig. 3B and C).

## 4 Discussion

In this study, the incidence rate of POCD was 28.8% at 7th day postoperatively and the main type of POCD was related with decreased speed of mental processing. The lab results suggested that there were significantly higher plasma levels of cortisol in patients with POCD at 1 week postsurgery. Moreover, we found that POCD patients displayed higher postoperative expression of FKBP51 instead of GR in leukocytes. To the best of our knowledge, this is the first clinical experiment demonstrating the correlation between FKBP51 and POCD.

Inflammation response plays a key role in the pathogenesis of POCD. Peripheral inflammation due to surgical trauma and the release of accompanying systemic inflammatory mediators have been shown to influence inflammatory processes of the central nervous system.^[11–13]^ Animal studies indicated that proinflammatory cytokines, such as interleukin 1β and tumor necrosis factor-α, play a pivotal role in mediating surgery-induced neuroinflammation.^[14]^ Inflammation response induced by surgery is usually transient and self-limited in most patients. However, POCD patients show enhanced inflammation response after surgery. Researches in animals and humans suggest that nonsteroidal anti-inflammatory drugs can inhibit inflammation and alleviate cognitive dysfunction.^[15,16]^ It is curious that dexamethasone, an anti-inflammatory agent, did not reduce the risk of POCD after surgery.^[17,18]^ Besides, it has been suggested that POCD patients presented higher cortisol levels after surgery.^[19]^ We suspected that GR signaling occurred disturbance in POCD patients and detected the expression level of GR. Our previous research indicates that the aberrant methylation of the GR gene promoter reduces the expression of the GR gene and facilitates exaggerated inflammatory responses.^[20]^ Nevertheless, in this study, no significant difference in expression levels of GR was found between groups POCD and non-POCD patients although POCD patients presented higher cortisol levels. This find indicated the cause of abnormal cortisol levels in POCD patients is not GR expression alterations but other factors, such as the dysfunction of GR-signaling transmission.

FKBP51, a key modulator of GR, modulates the stress response by antagonizing GR and regulating its sensitivity.^[6]^ FKBP51 presence in the protein complex induces a conformational change of the ligand-binding pocket that reduces GR hormone binding affinity. FKBP51 also prevents the nuclear translocation of the GR complex. High expression of FKBP51 can disturb GR signaling and result in glucocorticoid resistance,^[7]^ which may weaken a patient's resilience against inflammation induced by surgical trauma. We found POCD patients presented significantly higher FKBP51 levels in leukocytes compared with non-POCD patients in this study. This explains why dexamethasone fails in the prevention of POCD. There is another noticeable phenomenon that VAS scores in POCD group were slightly higher than that in non-POCD group after surgery under the same PCA scheme. Postoperative acute pain is another potential risk factor for cognitive dysfunction.^[21]^ One study showed that FKBP51 drives chronic pain by modulating spinal glucocorticoid signaling.^[7,22]^ FKBP51 may aggravate postoperative pain in POCD patients.

This study had several limitations. First, sample size was relatively small due to strict inclusion and exclusion criteria. The focus of the study is to test FKBP51 and not the epidemiology. We wanted to diminish as much as possible all the potential confounders. Second, without the evaluation of sleep quality after surgery, we cannot exclude sleep as a confounding factor in the present study. The third limitation is the 12% loss to 7 days follow-up; however, we performed a per-protocol analysis. Last, the follow-up period is short. POCD maybe is not maximal at 7th day after surgery. In conclusion, our findings suggest an association between higher expression of FKBP51 and POCD in aged patients. FKBP51 may be a suitable target for the prevention of POCD. However, the exact role of FKBP51 in the pathogenesis of POCD requires further evidence.

## Author contributions

**Conceptualization:** Zheng-Liang Ma.

**Data curation:** Li-Wei Wang, Mei-Yan Zhou.

**Formal analysis:** Li-Wei Wang, Mei-Jun Zhu.

**Funding acquisition:** Yan Li, Zheng-Liang Ma.

**Investigation:** Sheng-Tao Wang, Zheng-Liang Ma.

**Methodology:** Li-Wei Wang, Mei-Jun Zhu, Mei-Yan Zhou.

**Project administration:** Li-Wei Wang, Yan Li, You-Jia Yu, Zheng-Liang Ma.

**Resources:** Sheng-Tao Wang, Zheng-Liang Ma.

**Supervision:** Zheng-Liang Ma.

**Writing – original draft:** Li-Wei Wang.

**Writing – review & editing:** Zheng-Liang Ma.
